# Self-rated health after stroke: a systematic review of the literature

**DOI:** 10.1186/s12883-019-1448-6

**Published:** 2019-09-07

**Authors:** Érika de Freitas Araújo, Ramon Távora Viana, Luci Fuscaldi Teixeira-Salmela, Lidiane Andrea Oliveira Lima, Christina Danielli Coelho de Morais Faria

**Affiliations:** 1 Graduate Program in Rehabilitation Sciences of the Universidade Federal de Minas Gerais, (UFMG), Belo Horizonte, MG Brazil; 2 Department of Physical Therapy, Universidade Federal de Minas Gerais, (UFMG), Av. Antonio Carlos, 6627, Campus Pampulha, Belo Horizonte, MG ZIP code 31270-901 Brazil; 3 Department of Physical Therapy, Universidade Federal do Ceará (UFC), Fortaleza, Ceará Brazil

**Keywords:** Self-rated health, Perceived health, Self-assessment, Health status, Stroke, Cerebrovascular disorders, Review

## Abstract

**Background:**

Self-rated health (SRH) allows for comparison and identification of the health status of various populations. The aim of this study was to conduct a systematic review of the literature to expand the understanding of SRH after stroke.

**Methods:**

This systematic review was registered with PROSPERO (CRD42017056194) and conducted according to PRISMA guidelines. Studies published until December 2018 that evaluated the SRH of adults with stroke were included.

**Results:**

Of the 2132 identified studies, 51 were included. Only four studies had experimental designs (7.8%). In 60.7% of the studies, SRH was assessed by variations on direct questions (i.e., general and comparative SRH). Analog visual scales and quality of life instruments were also used to evaluate SRH, but there is no consensus regarding whether they are appropriate for this purpose. The results of cross-sectional and longitudinal studies revealed significant associations between poor SRH and stroke as well as between SRH, function, and disability. The power of SRH to predict stroke mortality is still uncertain. Two interventions (a home-based psychoeducational program concerning stroke health care and family involvement in functional rehabilitation) effectively improved SRH.

**Conclusions:**

Direct questions are the most common method of evaluating SRH after stroke. Studies reported significant associations between the SRH of individuals with stroke and several relevant health outcomes. However, few experimental studies have evaluated SRH after stroke. Interventions involving health education and family involvement had a significant impact on SRH.

## Background

Self-rated health (SRH) is a simple measure of how individuals evaluate their own health status [1, 2] recommended by the World Health Organization (WHO) [3]. The most common type of SRH evaluation, which asks simple and direct questions [4, 5], is useful within both clinical and research contexts [1] because it features reduced observation bias and lower costs related to measurement and data collection. This facilitates data analysis [6] and aligns with the principles of client-centered practice [6, 7]. SRH evaluations measure individuals’ perceptions of their own health and are therefore dependent on individuals’ pre-existing concept of health and context [4]. However, it is believed that people can synthesize a large amount of information about themselves in response to direct questions as part of an SRH evaluation [8].

Health status, quality of life (QoL), and health-related quality of life (HRQoL) are often used interchangeably [9]. According to the WHO, QoL refers to “individuals’ perceptions of their positions in life within the context of their culture and value systems in which they live, and in relation to their goals, expectations, standards, and concerns,” and it is “the product of the interplay between social, health, economic, and environmental conditions, which affect human and social development” [9]. In addition, the WHO defines health as “a state of a complete physical, mental, and social well-being, and not merely the absence of disease or infirmity” [9]. Therefore, compared to SRH, which is a measure of health status, QoL is a more comprehensive construct that covers all aspects of life. HRQoL is not clearly defined, but it is related to the way health affects QoL [10]. Despite their differences, these three constructs have similar characteristics; they are multidimensional, self-reported, and involve physical, mental, and social aspects of individuals’ lives [9, 10].

SRH has been considered a valuable outcome in studies with various objectives and populations. It has been demonstrated to have a significant association with the risk of diseases, such as depression [2] and type 2 diabetes [11], and/or decreased activity performance [1] in both adults [2, 11] and the elderly [1]. Recently, there have been several systematic reviews of the literature examining SRH with the elderly [12, 13], indigenous people [14], and adults in general [15]. Systematic reviews of the associations between SRH and relevant outcomes, such as mortality [16, 17], can also be found. However, no systematic literature reviews focus on the SRH of individuals with important, complex health problems, such as stroke.

Stroke is the leading cause of serious long-term disability, and it accounts for most of the global burden of disease [18]. Globally, the lifetime risk of stroke is 24.9% [18]. An estimated 7 million people suffered from a stroke worldwide between 2013 and 2016, and of those, about 1,806,000 were left with some type of disability [19]. One year after a stroke, 57% of people need assistance with daily living activities [20]. Therefore, health indicators of subjects with stroke, such as SRH, are of great clinical utility.

SRH has been associated with demographic, psychological, physical, and social factors in stroke patients [21] as well as increased risk of death [22] and the development of stroke in older adults without history of the disease [22]. Among elderly people who suffered from a stroke, poor SRH is related to reduced social interactions and limited mobility outside the home [21].

Due to the importance of SRH evaluations, the general aim of this study was to conduct a systematic literature review to expand the understanding of SRH after stroke. The specific aims were to (a) describe how SRH has been assessed and used, (b) synthesize previously reported results, and (c) verify the effects of interventions on the SRH of individuals who suffered from a stroke.

## Methods

This systematic review followed the Preferred Reporting Items for Systematic Review and Meta-Analysis (PRISMA) guidelines [23, 24], and it was registered in the International Prospective Register of Systematic Reviews (PROSPERO; CRD42017056194). All steps were performed by two independent examiners, and a third examiner was involved in cases of lack of consensus.

This review included studies that assessed SRH in individuals ≥18 years of age who suffered from a stroke. The WHO’s definition of a stroke was adopted [25]. Of the studies with mixed populations, only those that separately reported the SRH results of individuals with stroke were included. All studies published until December 2018 in any language, except for theses or dissertations, case series, or case studies, were analyzed.

Searches were conducted in the following electronic databases: Medical Literature Analysis and Retrieval System Online (MEDLINE), Physiotherapy Evidence Database (PEDro), Latin American and Caribbean Health Sciences Literature (LILACS), and Scientific Electronic Library Online (SCIELO). An initial search strategy was created for MEDLINE and then was adapted to the other databases. The search strategy for stroke published in a recent systematic review of the Cochrane Database of Systematic Reviews [26] was elaborated upon by the authors with terms that were used in previous systematic reviews of the same outcome of interest (i.e., SRH; see Appendix for the search terms) [3, 17, 22, 27].

All studies found in the electronic databases were screened based on their titles and abstracts. Those that clearly did not meet the eligibility criteria were excluded. Then, the full texts of the remaining studies were analyzed to determine whether they met the eligibility criteria. A manual search in the references section was also performed in the included studies. The methodological quality of the clinical trials was evaluated using the PEDro scale [28], and the risk of bias in quasi-experimental studies was evaluated by the Transparent Reporting of Evaluations with Nonrandomized Designs (TREND) [29]. Information that was relevant to the objectives of this systematic review was extracted using a structured form developed based in a prior study that contained the following information: author/year, study design, objective, sample characteristics, instrument/question used to evaluate SRH, response items, SRH operationalization, statistical analysis, and conclusions regarding SRH.

## Results

**Fig. 1:**
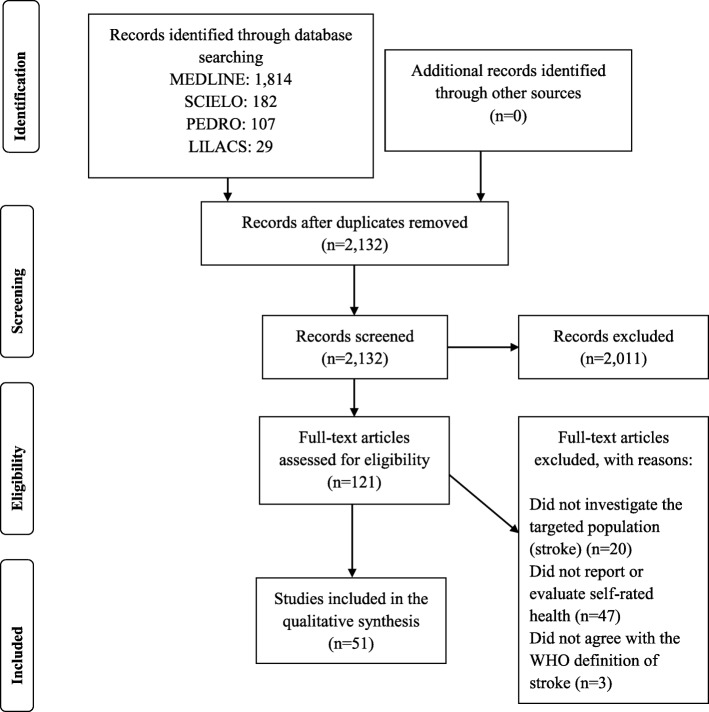
Flow diagram of the study selection process. Adapted PRISMA flow diagram (2009). LILACS = Latin American & Caribbean Health Sciences Literature, MEDLINE = Medical Literature Analysis and Retrieval System Online, n = number of studies, PEDro = Physiotherapy Evidence Database, SCIELO = Scientific Electronic Library Online, WHO = World Health Organization

Of the 2132 identified studies, 51 were included in this review (Fig. 1). Of these, 49% (*n* = 25) included subjects from European countries [5, 21, 22, 30–49] and 27.5% (*n* = 14) included subjects from North America [50–63]. The majority had a longitudinal design (*n* = 30, 58.8%) [5, 22, 30–32, 34, 36–42, 46–48, 52, 56–58, 60, 61, 63–69]. Only three longitudinal studies (10%) [34, 62, 65] were randomized clinical trials, and one (3.3%) [66] was a quasi-experimental study.

The sample size of the included studies ranged from 19 [67] to 104,876 [68]. Of the studies that reported the sex of the participants (*n* = 32, 62.7%) [5, 21, 22, 31–36, 38–42, 44, 46–49, 52–54, 57–60, 65, 67, 68, 70, 71], 23 (71.8%) [5, 31–33, 35, 38, 40–42, 44, 46–49, 55, 57, 58, 60, 65, 67, 68, 70, 71] used a male-dominated sample. In the 23 studies (45%) that provided information on age [5, 21, 22, 32–34, 36, 38, 39, 41, 42, 44, 46, 48, 52, 55, 57–60, 65, 67, 68], the mean age of the participants ranged from 43 ± 14 [45] to 77 ± 7 years [54]. Stroke was predominantly ischemic and in a chronic phase in all studies that provided this information (*n* = 12, 23.5%; Table 1) [31–33, 39, 40, 42, 47, 48, 55, 57, 67, 68].

**Table 1:** Studies characteristics regarding the sample and self-rated health (*n* = 51)

Study/ Country	Stroke sample characteristics	SRH measure	SRH operationalization
Ho, 2018 [72] / Taiwan	*n* = 98	Excellent, Good, Average, Not so good, Poor	Done
Jönsson et al., 2018 [41] / Sweden	*n* = 145 (W = 41%, M = 59%) Mean age (years) = 66.2, range = 17.5–87.1 Cerebral infarction = 87%, Intracerebral hemorrhage = 7%, Subarachnoid hemorrhage = 5.5%, Undefined = 0.5% Acute and chronic stroke (16 months and 10 years)	SF-36 (first question)	Not done
Kim, Lee, 2018 [71] / Korea	*n* = 4322 (W = 49%, M = 51%) Women’s age (years): 19–49 = 2.4%, 50–64 = 19.1%, 65–79 = 61.9%, ≥80 = 16.6% Men’s age (years): 19–49 = 4.5%, 50–64 = 24.5%, 65–79 = 57.7%, ≥80 = 13.3%	Good, Fair, Poor	Not done
Song et al., 2018 [73] / China	*n* = 8884	Excellent, Good, Fair, Poor Better, About the same, Worse, Don’t know	Done
Dong et al., 2018 [69] / China	*n* = 7572	Excellent, Good, Fair, Poor, Better, About the same, Worse, Don’t know	Not done
Mavaddat et al., 2018 [42] / United Kingdom	*n* = 28 (W = 32%, M = 68%) Age range (years) = 47–86	Excellent, Good, Fair, Poor, Very poor	Not done
Vogelsang, 2017 [55] / United States of America	*n* = 948	Better, Same, Worse	Not done
Guerard et al., 2016 [54] / United States of America	Chronic stroke	Much worse, Slightly worse, About the same, Slightly better, Much better	Done
Larsen et al., 2016 [44] / Denmark	*n* = 590 (W = 36%, M = 64%) Age (years): ≤49 = 38%, 50–60 = 62% Ischemic = 86%, Intracerebral hemorrhage = 11%, Unspecified = 3%	SF-12	Not done
Larsen et al., 2016 [40] / Denmark	*n* = 2414 (W = 39.2%, M = 60.8%) Age (years): ≤59 = 27%, 60–69 = 35%, ≥70 = 38% Ischemic = 87%, Iintracerebral hemorrhage = 9%, Unspecified = 4% Subacute to chronic stroke (3–6 months)	SF-12	Not done
Mavaddat et al., 2016 [22] / United Kingdom	*n* = 776 (W = 51.5%, M = 48.5%) Mean age (years) = 76.2 Chronic stroke	Excellent, Good, Fair, Poor	Done
Patterson, Sibley, 2016 [57] / Canada	*n* = 1892 (W = 52%, M = 48%) With arthritis = 53.4%, Without arthritis = 46.6% Age (years): 50–54 = 4.4%, 55–59 = 8.1%, 60–64 = 13.1%, 65–69 = 14.1%, 70–74 = 15.2%, 75–79 = 16.2%, ≥80 = 28.9%	Excellent, Very good, Good, Fair, Poor	Done
Arokiasamy et al., 2015 [74] / China, Ghana, India, Mexico, Russia and South Africa	Chronic stroke	Very good, Good, Moderate, Bad, Very bad	Done
Egan et al., 2015 [52]/ Canada	*n* = 67 (W = 41.8%, M = 58.2%) Mean age ± SD (years) = 64.8 ± 13.3, range = 33–88 Chronic stroke	Excellent, Very good, Good, Fair, Poor	Not done
Sand et al., 2015 [39]/ Norway	*n* = 327 (W = 37.3%, M = 62.7%) Vision problem = 25.4%, Mean age ± SD (years) = 71.8 ± 14.3 Normal vision = 74.6%, Mean age ± SD (years) = 66.5 ± 12.4 Chronic stroke (6 months)	Very good, Good, Neither good nor bad, Bad, Very bad	Not done
Theme Filha et al., 2015 [77]/ Brazil	*n* = 918 Chronic stroke	Very good, Good, Moderate, Bad, Very Bad	Done
Waller et al., 2015 [38] / Sweden	*n* = 115	Better, Worse, Similar	Not done
Arruda et al., 2015 [75] / Brazil	*n* = 38	Excellent, Very good, Good, Fair, Poor	Not done
Mavaddat et al., 2014 [70] / United Kingdom	*n* = 342 (W = 40.9%, M = 59.1%) Chronic stroke	Excellent, Good, Moderate, Poor	Done
Ostwald et al., 2014 [62] / United States of America	*n* = 159 (W = 25.2%, M = 74.8%) Control group = 50.3%, Mean age ± SD (years) = 65.75 ± 9.26 Experimental group = 49.7%, Mean age ± SD (years) = 66.98 ± 9.04 Chronic stroke (1 year)	SF-36 (first question)	Not done
Shen et al., 2014 [66] / China	Not reported	Better, Normal, Worse	Not done
Chang et al., 2013 [67] / South Korea	*n* = 19 (W = 47.4%, M = 52.6%) Mean age ± SD (years) = 74.47 ± 4.64, range = 67–82 Ischemic = 68.4%, Hemorrhagic = 21.1%, Combined = 10.5% Chronic stroke (1 year)	Visual analog scale 1 (not healthy at all) to 10 (very healthy)	Not done
Fernández-Ruiz et al., 2013 [43]/ Spain	*n* = 203	1 (Very good, Good, Fair, Poor, Very poor) 2 (Much better, Better, Similar, Worse, Much worse)	Done
Varela et al., 2013 [76] / Brazil, Mexico, Uruguay, Chile and Venezuela	*n* = 120 With COPD = 20%, Without COPD = 80%	Excellent, Very good, Good, Fair, Poor	Not done
Latham, Peek, 2013 [53] / United States of America	*n* = 209 Chronic stroke	5 = Excellent, 4 = Very good, 3 = Good, 2 = Fair, 1 = Poor	Not done
Mavaddat et al., 2013 [21] / United Kingdom	n = 776 (W = 51.5%, M = 48.5%) Mean age (years) = 76.2, 64–74 = 40.2%, 75–84 = 48.3%, ≥85 = 11.5% Chronic stroke (5 years)	Excellent, Good, Fair, Poor	Done
Cerniauskaite et al., 2012 [37] / Italy	*n* = 111 (W = 46%, M = 54%) Mean age ± SD (years) = 57.8 ± 14.4, range = 22–86 Chronic stroke (mean 5.4 years)	Better Health, Unchanged Health, Worse Health	Not done
Prlić et al., 2012 [35]/ Croatia	*n* = 161 (W = 49%, M = 51%) Mean age (years) = 69.89, range = 35–98 Ischemic = 90%, Hemorrhagic = 10% Acute stroke	SF-36	Not done
Foraker et al., 2011 [56] / United States of America	*n* = 809 (W = 55.3%, M = 44.7%) Mean age (years) = 54.7 Acute stroke	Excellent, Good, Fair, Poor, Death	Not done
Welin et al., 2010 [34] / Sweden	*n* = 163 (W = 38.7%, M = 61.3%) Control group = 50.3%, Mean age ± SD (years) = 69.6 ± 11.7 Experimental group = 49.7%, Mean age ± SD (years) = 71.2 ± 9.9 Hemorrhagic = 12.3%	Scale 1 (Excellent) to 5 (Poor)	Done
Asplund et al., 2009 [68] / Sweden	*n* = 104,876 (W = 47.5%, M = 52.5%) Mean age (years) = 74.4 Ischemic = 86.3%, Hemorrhagic = 9.5%, Unspecified = 4.2% Subacute Stroke (3 months)	Very good health, Fairly good health, Fairly poor health, Very poor health	Done
Boyington et al., 2008 [63] / United States of America	*n* = 580 (W = 61.4%, M = 38.6%) Mean age ± SD (years) = 76.61 ± 7.35 years, ≥75 = 58.3%, < 75 = 41.7%	Excellent, Good, Fair, Poor	Done
Goebeler et al., 2007 [36]/ Finland	*n* = 41 Stroke diagnosis in medical records = 70.7%, Self-reported stroke = 29.3% Chronic Stroke	Very good, Fairly good, Fairly poor, Very poor	Not done
Olsson, Sunnerhagen, 2007 [33] / Sweden	*n* = 50 (W = 48%, M = 52%) Ischemic = 70%, Hemorrhagic = 30%	EQ*therm*	Not done
Skånér et al., 2007 [32] / Sweden	*n* = 145 (W = 52.4%, M = 47.6%) Mean age ± SD (years) = 73.3 ± 11.8 Ischemic = 77.9%, Hemorrhagic = 6.2%, Unspecified = 15.9% Chronic stroke (1 year)	Very good, Rather good, Neither good nor poor, Rather poor, Poor	Not done
Martins et al., 2006 [46] / Portugal	*n* = 273 (W = 45.4%, M = 54.6%) Mean age ± SD (years) = 69.2 ± 11.8, range = 40–100 Ischemic = 83%, Hemorrhagic = 11%, Unspecified = 6%	COOP/WONCA	Not done
Olsson, Sunnerhagen, 2006 [45]/ Sweden	*n* = 52 (W = 46.2%, M = 53.8%) Mean age ± SD (years) Ischemic = 52 ± 7.4, Cerebral infarction = 44.4 ± 17.8, Subarachnoidal bleeding =43.3 ± 13.8 Ischemic = 71.2%, Hemorrhagic = 28.8% Chronic stroke (mean 6 months, range = 22 days-15 months)	EQ*therm*	Not done
Salbach et al., 2006 [59]/ Canada	*n* = 86 (W = 35%, M = 65%) Mean age (years) = 71.5, range = 38–91 Ischemic = 86%, Hemorrhagic = 14% Chronic stroke (1 year)	EQVAS	Not done
Salbach et al., 2006 [61] / Canada	*n* = 89 (W = 37%, M = 63%) mean age ± SD (years) = 72 ± 11, range = 38–91 Ischemic = 84.3%, Hemorrhagic = 15.7% Subacute stroke (2 months)	EQVAS	Not done
Emmelin et al., 2003 [31] / Sweden	*n* = 473 (W = 41.6%, M = 58.4%) Mean age (years) = 54.9 Acute stroke	Very good rather good, Neither good nor bad, Rather bad, Bad	Done
Hillen et al., 2003 [5]/ United Kingdom	*n* = 561 (W = 47%, M = 53%) Mean age ± SD (years) = 69.4 ± 13.7 Hemorrhagic = 15.7% Subacute stroke (3 months)	1 (Excellent, Very good, Good, Fair, Poor) 2 (Much better, Somewhat better, About the same, Somewhat worse, Much worse)	Not done
Otiniano et al., 2003 [58] / Mexico	*n* = 190 (W = 52.6%, M = 47.4%) Diabetes + Stroke = 40%, No diabetes + Stroke = 60% Age (years): 65–74 = 48%, ≥75 = 52% Chronic stroke	Excellent, Good, Fair, Poor	Done
Muntner et al., 2002 [60] / United States of America	*n* = 1003	1 = Excellent, 2 = Very good, 3 = Good, 4 = Fair, 5 = Poor	Not done
Han et al., 2001 [51] / United States of America	*n* = 591 Chronic stroke	1 (1 = Excellent, 2 = Very good, 3 = Good, 4 = Fair, 5 = Poor) 2 (1 = Better, 3 = Same, 5 = Worse)	Not done
Bugge et al., 2001 [47] / United Kingdom	*n* = 153 (W = 51%, M = 49%) Mean age (years) = 70.6, range = 35–93 Acute stroke	SF-36	Not done
Anderson et al., 2000 [65] / Australia	*n* = 86 (W = 44.2%, M = 55.8%) Control group = 51.2%, Experimental group = 48.8% Mean age (years) = 71.5 Acute stroke	SF-36	Not done
Hoeymans et al., 1999 [30] / Netherlands	*n* = 66 Chronic stroke	Healthy, Rather healthy, Moderately healthy, Not healthy	Done
Deane et al., 1996 [49] / United Kingdom	*n* = 27 (W = 70.4%, M = 29.6%) Mean age (years) = 51, < 65 = 85.2%, ≥65 = 14.8%, range = 33–72 Chronic stroke (6 months)	SF-36	Not done
Tuomilehto et al., 1995 [48] / Finland	*n* = 201 (W = 49.8%, M = 50.2%) Age (years): ≤64 = 36.8%, ≥65 = 63.2% Chronic stroke (14 years)	Sum of scores from 1 to 4 in the items: patient’s own perceived health, frequency of symptoms, and the frequency of occasions when they had been worried about their health (last month)	Done
Tsuji et al., 1994 [64] / Japan	*n* = 34 (deaths for stroke)	Excellent, Good, Fair, Poor	Done
Pope, 1988 [50] / United States of America	*n* = 138	Excellent, Good, Fair, Poor	Not done

Regarding the SRH assessment, 72.5% of the studies (*n* = 37) [5, 21, 22, 30, 33, 37, 39, 43–46, 49–52, 59–64, 66, 68–71, 73–77] used general direct questions (*n* = 21, 67.7%) [5, 30, 33, 37, 39, 43, 46, 49, 51, 59–61, 64, 68–71, 73, 74, 76, 77] or comparative direct questions (*n* = 15, 48.4%) [5, 21, 22, 30, 44, 45, 50–52, 62, 63, 66, 69, 73, 75]. Comparative SRH referred to patients’ current health status in comparison to an earlier period [5, 37, 43, 51, 54–56, 66] or to people of the same age [21, 22, 38, 43, 50, 56, 66, 69, 73, 75]. SRH was also assessed by the full (SF-36) [35, 47, 49, 62, 65] and short (SF-12) versions of the Short-Form Health Survey questionnaire [40, 44]; the visual analogue scale [33, 45, 59, 61, 67]; and the EuroQol 5D (EQ5D) [33, 45, 59, 61].

### Results and conclusions of the cross-sectional studies

In 11 of the 21 cross-sectional studies (52.4%) [21, 36, 38, 54, 57, 70, 72, 73, 75, 77], poor SRH was significantly associated with poor outcomes, such as reduced mobility and limitations in activities of daily living (ADL) [63], decreased functionality [37], poorer affective-emotional and social state [46], poor marital status [72], and the presence of other health conditions [51, 71] (Table 2).

**Table 2:** Statistical analyses and conclusions regarding self-rated health in people with stroke – cross sectional studies (*n* = 21)

Study	Inferential statistical analysis	Conclusions about self-rated health
Ho, 2018 [72]	Multinomial logistic regression model	Stroke were found to be a significant predictive factor related to worse SRH in elder widowed people
Kim, Lee, 2018 [71]	Multivariate logistic regression model	Suicidal ideation was significantly more common among stroke survivors with poor SRH compared with good SRH for both genders, male and female
Song et al., 2018 [73]	Multivariate logistic regression model	Stroke was the most important factor associated with worse age comparative SRH among total population, rural residence and male individuals
Mavaddat et al., 2018 [42]	Qualitative Thematic analysis	SRH after a stroke is based in a multidimensional appraisal and reflect the combination of of physical, psychological and social influences, from past and future perceptions of health.
Guerard et al. 2016 [54]	Multinomial logistic regression model	Significant association between stroke episode and SRH
Patterson, Sibley 2016 [57]	Multiple logistic regression model	In people with stroke, the risk of arthritis is higher than in healthy people and the association of these two comorbidities was related to poor SRH
Arokiasamy et al. 2015 [74]	Multinomial logistic regression model	Not reported
Theme Filha et al. 2015 [77]	Multiple logistic regression model	Stroke was the chronic non-communicable disease with the highest proportion of bad answers in SRH
Waller et al. 2015 [38]	Ordinal logistic regression model	Stroke was associated to a worse age comparative SRH
Mavaddat et al. 2014 [70]	Multiple logistic regression model	Poor SRH was associated to stroke especially with other comorbidities
Arruda et al. 2015 [75]	Multiple logistic regression model	Poor SRH was associated to stroke in adults
Varela et al. 2013 [76]	Chi square test	More than a half of people with COPD, who had a stroke showed good or excellent SRH
Mavaddat et al. 2013 [21]	Multiple logistic regression model	Social aspects and diabetes showed to be related to poor SRH in older individuals with stroke
Cerniauskaite et al. 2012 [37]	Pearson correlation coefficient	SRH had a strong correlation with functionality in people with stroke
Boyington et al. 2008 [63]	Multiple logistic regression model	SRH in people with stroke had no differences related to skin color. However, when these people present limitations in ADL and mobility, SRH become more important for whites than to blacks
Goebeler et al. 2007 [36]	Chi square test	In individuals over than 90 years old and with stroke, SRH was poor
Salbach et al. 2006 (1) [59]	Cronbach alpha measure of internal consistency	Not reported
Martins et al. 2006 [46]	Correlation measures	SRH showed a strong correlation with the emotional state, ability to perform ADL and social life
Han et al. 2008 [51]	Structure equation modeling	In elder, the presence of other health condition beyond stroke had more influence in SRH evaluation
Tuomilehto et al. 1995 [48]	Not done	85% of the respondents 14 years post stroke, reported good or satisfied health. Although, one third showed poor functional capacity due to permanent sequelae of the stroke
Pope, 1988 [50]	Multiple logistic regression model	Poor SRH was associated to severe chronic health conditions like stroke

*ADL* activities of daily living, *COPD* chronic obstructive pulmonary disease

### Results and conclusions of the longitudinal observational studies

In the six longitudinal observational studies (23.1%) [5, 22, 30, 39, 40, 60], poor SRH was associated with stroke. One study (3.8%) [39] reported this relationship specifically for individuals who reported post-stroke visual impairments. The predictive power of SRH for stroke mortality could not be confirmed since significant results were observed in two studies [43, 66] but not in two others [22, 64]. SRH was associated with morbidity, especially after a stroke [31, 53, 69], and with return to work and post-stroke stability [44]. A combination of diabetes and stroke was strongly associated with poor SRH [58]. Furthermore, improvements in balance self-efficacy were associated with improvements in functional walking capacity, which in turn led to increased SRH [61] (Table 3).

**Table 3:** Statistical analyses and conclusions about self-rated health in people with stroke – longitudinal observational studies (*n* = 26)

Study	Inferential statistical analysis	Conclusions about self-rated health
Jönsson et al., 2018 [41]	Wilcoxon test	There was no significant difference in SRH between stroke survivors in acute phase (16 months) and in a long term (10 years)
Dong et al., 2018 [69]	Cox proportional hazards model	General and age comparative SRH were significantly associated with an increased risk of first-ever stroke and recurrent stroke in Chinese adults
Vogelsang, 2017 [55]	Logistic regression model	Stroke is associated with improvement in comparative SRH but not with retrospectively reported SRH
Mavaddat et al., 2016 [22]	Cox proportional hazards model	There is a small but significant independent relationship between poor SRH and stroke incidence. However there is no relationship between SRH and stroke mortality in the short or longer term in the older population. In older people with a history of stroke, there is no relationship between SRH and stroke outcomes
Larsen et al., 2016 [44]	Logistic regression model	SRH 3 months post-stroke and stroke severity were found to be strongly associated with return to work and subsequent work stability after stroke
Larsen et al., 2016 [40]	Linear regression model	Stroke patients rated their health 3 months post stroke lower on all SF-12 scales than the general Danish population
Egan et al., 2015 [52]	Bivariate correlations, Linear regression model, Generalized estimating equation	Better perceived health was associated with higher scores in the instrument of participation evaluation, RNLI
Sand et al., 2015 [39]	Logistic regression model	Patients reporting vision problems rated their own general health as significantly poorer
Shen et al., 2014 [66]	Cox proportional hazards model	The association of age-comparative SRH with death from stroke varied by sex, with the association stronger for men than women
Latham, Peek, 2013 [53]	Cox proportional hazards model	SRH is a significant independent predictor of global morbidity onset and cause-specific morbidity onset, including stroke, excluding cancer, even after controlling for important sociodemographic characteristics, health care access and utilization, and risk factors
Fernández-Ruiz et al., 2013 [43]	Cox proportional hazards model	Age-comparative SRH was considered a strong predictor of stroke mortality
Prlić et al., 2012 [35]	Friedman test	Women with stroke rated their physical and mental health (SF-36) worse than men with stroke
Foraker et al., 2011 [56]	Regression model	There was a decline statistically significant in SRH, both pre- and post-disease, in different incident disease types (cardiac revascularization procedure, myocardial infarction, lung cancer, heart failure) except for stroke
Asplund et al., 2009 [68]	Multinomial logistic regression model	The minority of patients with stroke and poor SRH showed dissatisfaction with health care and social services at large
Olsson, Sunnerhagen, 2007 [33]	Spearman correlation coefficient	Stroke patients age 18 to 60 years at the time of acute stroke who received 6–8 weeks of DHR post stroke were able to maintain their levels of SRH 2 years after being discharged from DHR to their own homes, especially for men
Skånér et al., 2007 [32]	Not done	The majority of patients rated their health as rather good or very good at 3 and 12 months after stroke
Salbach et al., 2006 (2) [61]	Spearman correlation coefficient	Enhancing balance self-efficacy in addition to functional walking capacity is expected to enhance physical function and perceived health status to a greater extent than enhancing functional walking capacity alone
Olsson, Sunnerhagen, 2006 [45]	Linear regression model	After 6 to 8 weeks of DHR after acute treatment for stroke there were improved physical and cognitive functions, and improved SRH
Emmelin et al., 2003 [31]	Univariate and multivariate logistic regression model	Self-rated ill-health independently increases the risk of stroke, specifically for men, and that the interaction effect between SRH and biomedical risk factor load is greater for men than for women
Hillen et al., 2003 [5]	Wilcoxon test, Logistic regression model	Patients reporting a health transition to “much worse” 3 months after stroke have an increased risk of disability at 1 year and decreased chances to survive free of stroke recurrence over the next 5 years
Otiniano et al., 2003 [58]	Chi square test, Logistic regression model	Diabetes and stroke in combination is strongly associated with a higher risk of disabilities, poor SRH, and higher 5-year mortality rates than persons without these diseases, regardless of the presence of other conditions
Muntner et al., 2002 [60]	Not done	Self-reported “health in general” was worse among those with a history of stroke compared with those without a history of stroke for all three time periods (1971–1975, 1976–1980 e 1988–1994)
Bugge et al., 2001 [47]	Wilcoxon test, Multiple linear regression model	Although, stroke patients perceived their health to be worse than the general population in many dimensions of SF-36, they perceived their “General health” more positively
Hoeymans et al., 1999 [30]	Logistic regression model	Stroke was the disease that resulted in the largest loss in SRH in patients, followed by respiratory symptoms, coronary heart disease, musculoskeletal complaints, and diabetes
Deane et al., 1996 [49]	Not done	Not reported
Tsuji et al., 1994 [64]	Cox proportional hazards model	SRH was significant associated to death for cancer but not for stroke or heart disease

*DHR* day hospital rehabilitation, *RNLI* Reintegration to Normal Living Index, *SF-12 and SF-36* Short Form Health Survey 12 and 36

### Results and conclusions of the longitudinal experimental studies

According to the PEDro scale, scores of the methodological quality of three experimental longitudinal trials [34, 62, 65] ranged from six [34] to eight [65], which is considered good [78]. The quasi-experimental study [67] scored 13 points out of 22 on the TREND scale (Table 4). Only the study of Ostwald et al. [62], which compared the efficacy of a home-based psychoeducational program concerning stroke health care to mailed information on stroke prevention, showed that improvements in SRH favored the experimental group [62].

**Table 4:** Quality analyses of the longitudinal studies – PEDro (*n* = 3) and TREND scale (*n* = 1)

Criteria of PEDro scale																							
Study	P1		P2		P3		P4		P5		P6		P7		P8		P9		P10		P11	T (0 a 10)	
Ostwald, et al., 2014 [62]	Y		Y		Y		Y		N		N		Y		N		Y		Y		Y	7	
Welin, et al., 2010 [34]	Y		Y		Y		Y		N		N		N		Y		N		Y		Y	6	
Anderson et al., 2000 [65]	Y		Y		Y		Y		N		N		Y		Y		Y		Y		Y	8	
Criteria of TREND statement																							
Study	T1	T2	T3									T4								T5			T
	T1.1	T2.1	T3.1	T3.2	T3.3	T3.4	T3.5	T3.6	T3.7	T3.8	T3.9	T4.1	T4.2	T4.3	T4.4	T4.5	T4.6	T4.7	T4.8	T5.1	T5.2	T5.3	
Chang et al., 2015 [65, 67]	Y	Y	Y	Y	Y	N	N	N	N	N	Y	N	Y	Y	NA	Y	Y	N	N	Y	Y	Y	13

P1 = eligibility criteria, P2 = randomly allocated, P3 = allocation concealed, P4 = similar groups at baseline, P5 = blinding subjects, P6 = blinding therapists, P7 = blinding assessors, P8 = losses < 15%, P9 = intention to treat analysis, P10 = results of between-group statistical comparisons reported, P11 = point measures and measures of variability reported, T1 = Title and Abstract, T1.1 = Information about allocation, target population and structured abstract; T2 = Introduction, T2.1 = Scientific background and explanation of rationale, T3 = Methods, T3.1 = Eligibility criteria for participants, method of recruitment, recruitment setting; T3.2 = Details of the interventions, T3.3 = Specific objectives and hypotheses, T3.4 = Clearly defined primary and secondary outcome measures, information on validated instruments; T3.5 = Sample size determined, T3.6 = Method used to assign units to study conditions, T3.7 = Blinding subjects, therapists and assessors; T3.8 = Description of the smallest unit that is being analyzed to assess intervention effects, If the unit of analysis differs from the unit of assignment, the analytical method used to account for this; T3.9 = Statistical methods used, statistical software or programs used, methods for imputing missing data; T4 = Results, T4.1 = Flow of participants and description of protocol deviations, T4.2 = Periods of recruitment and follow-up, T4.3 = Baseline data, T4.4 = Baseline equivalence, T4.5 = Number of participants and indication of whether the analysis strategy was “intention to treat”, T4.6 = Each primary and secondary outcome and inclusion of null and negative findings, T4.7 = Ancillary analyses, T4.8 = Adverse events, T5 = Discussion, T5.1 = Interpretation of the results, T5.2 = Generalizability (external validity), T5.3 = Overall Evidence, T = total 0 a 22, *Y* Yes, *N* No, *NA* not applicable, *PEDro* Physiotherapy Evidence Database, *TREND* Transparent Reporting of Evaluations with Nonrandomized Design

The quasi-experimental study [67], which investigated the effects of family involvement in functional rehabilitation performed by a physiotherapist and a nurse at a rehabilitation center for post-stroke elderly patients, found significant improvements in SRH after the end of the intervention.

## Discussion

The present study performed a systematic review of the literature on SRH after stroke. Most of the studies employed general and comparative direct questions to assess SRH. The cross-sectional and longitudinal observational studies revealed significant associations between poor SRH, stroke, and other important health outcomes. In addition, SRH was reported to predict the occurrence of stroke. However, the association between SRH and stroke mortality remains unclear. To date, few studies have evaluated the effects of interventions on SRH. However, those that do exist found two types of interventions that effectively improve the SRH of individuals with stroke.

SRH was mostly evaluated by direct questions, although there was great variability in the structure of these questions and the response items. Jürges et al. [79] reviewed two versions of response items, the one recommended by the WHO (*Very Good*, *Good*, *Fair*, *Bad*, and *Very Bad)* and the European version (*Excellent*, *Very Good*, *Good*, *Fair*, and *Poor)*. Although some differences were found, both versions were highly correlated after the items were resized to allow for comparison [79]. Comparative questions mainly had three options, making direct comparison with general SRH evaluations difficult [4].

Studies investigating elderly people compared general and comparative direct questions but reported different results [80, 81]. One study, which investigated whether the reference point (i.e., people of the same age) would be a good predictor of mortality in the elderly, showed that comparative questions better predicted mortality in men [80]. However, the other study, which compared general and comparative questions among elderly individuals of the same age, found that general questions were better since the comparative questions are influenced by age [81]. To our knowledge, no study has compared general and comparative questions among stroke patients, and therefore there is no scientific information that can be used to determine the best method of assessing SRH.

The total scores of QoL/HRQoL instruments, such as the SF-36 and the SF-12, were used to evaluate SRH [35, 47, 49, 62, 65]. Although QoL, HRQoL, and health status have some similarities, they use different constructs [9]. Additionally, if the aim is to measure health status, specific questions should be used. Some QoL/HRQoL instruments have some SRH-specific questions, such as the first and second items of SF-36 [41]. However, the total scores of QoL/HRQoL instruments cannot be used as SRH measures. Therefore, the results of studies that assess SRH based on total scores [35, 47, 49, 62, 65] should be considered with caution.

The most common analog visual scale for SRH assessment is the EQ5D [82]. This scale is traditionally used to measure subjective phenomena, and it is easy and quick to apply, with excellent properties for measuring pain and QoL/HRQoL [83]. However, it is necessary to investigate whether its measurement properties are adequate for SRH evaluation [84]. Therefore, the results of the studies that use analog visual scales [33, 45, 59, 61] should also be interpreted with caution.

In most studies, poor SRH was associated with stroke. Since SRH is a measure and indicator of health status [3], this finding may reflect the health care needs of this population [75]. According to this review, studies have found strong associations between poor SRH and visual impairments [39], balance impairments [61], worse affective-emotional state [46], mobility deficits [63], limitations in ADL [63], worse function [37], reduced social life [46] and inability to return to work [44]. Additionally, using the Barthel index, Hillen et al. [5] reported that functional independence appeared to have a greater impact on SRH than other outcomes and was a strong predictor of health status in individuals with stroke [5].

Some studies did not find any association between poor SRH and stroke. Tuomilehto et al. [48] observed good or satisfactory SRH in individuals 14 years after a stroke episode. These findings could be explained by the duration of the disease; longer periods of time since the onset of stroke were associated with better acceptance of disability [85]. Most of the individuals investigated in prior studies with better SRH had mild disabilities [48] and higher survival rates [86]. In their qualitative study, Mavaddat et al. [42] reported that the severity of physical limitations alone did not influence perceptions of SRH; even individuals with severe morbidities could report good SRH. Similarly, Varela et al. [76] found an association between good SRH and patients with chronic obstructive pulmonary disease (COPD) who had suffered a stroke.

Many studies have demonstrated the predictive power of SRH for mortality and morbidities among various populations [8, 36, 69]. However, two studies employing a logistic regression model adjusted for sociodemographic factors and morbidities found that SRH did not predict mortality after stroke. Adjusting sociodemographic factors does not reduce the predictive ability of SRH measures, but the presence of morbidities, especially among respondents with poor SRH, tends to reduce predictive power [16].

Only two of the four experimental longitudinal studies found improvements in SRH [62, 67]. This outcome could be changed only by interventions targeting factors that observational studies found were related to SRH. The improvements in SRH produced by a home-based psychoeducational program concerning health care after stroke may be related to the educational level of the individuals [87]; understanding of the disease is an important factor affecting SRH [62]. Family involvement in functional rehabilitation also improved the SRH of individuals with stroke [67], and it is recognized to have strong psychological effects on therapy through physical and emotional support [4, 88].

SRH was used as an outcome less often in experimental longitudinal studies than in observational studies. Future experimental studies should examine SRH evaluation due to its importance and informative capacity for individuals’ health [3]. In addition, healthcare professionals should routinely assess SRH using both general and comparative simple and quick questions [4] in order to identify and summarize the health status of patients with stroke. Poor SRH may be used to identify priority patients since it is commonly related to disabilities, and it can be used to monitor patients who have not had a stroke but are at risk. Home-based psychoeducational programs as well as family involvement in functional rehabilitation at rehabilitation center for post-stroke may be used as interventions to improve SRH.

## Conclusions

Direct questions were the most commonly mentioned and recommended way to measure SRH in subjects with stroke. It is unclear whether a certain type of question is superior to others, but general SRH is most commonly used. Cross-sectional and longitudinal studies have shown significant associations between SRH and several relevant health outcomes, while few experimental studies have evaluated the efficacy of interventions for improving SRH after stroke.

## Data Availability

The datasets used and/or analysed during the current study are available from the corresponding author on reasonable request.

## Appendix

### Search strategy of MEDLINE (Pubmed)

1. Cerebrovascular disorders [mh] OR brain injuries [mh] OR hemiplegia [mh] OR paresis [mh] OR dystonia [mh]
2. Stroke [tw] OR poststroke [tw] OR post-stroke [tw] OR cerebrovasc* [tw] OR brain vasc* [tw] OR cerebral vasc* [tw] OR cva [tw] OR apoplex* [tw]
3. Brain [tw] OR cerebro* [tw] OR cerebra* [tw] OR cerebell* [tw] OR intracran* [tw] OR intracerebral [tw] OR vertebrobasilar [tw]
4. ischemi* [tw] OR ischaemi* [tw] OR infarct*[tw] OR thromboa* [tw] OR thrombob* [tw] OR thromboc* [tw] OR thromboe* [tw] OR thrombof* [tw] OR thrombog* [tw] OR thromboh* [tw] OR thromboi* [tw] OR thrombok* [tw] OR thrombol* [tw] OR thrombom* [tw] OR thrombon* [tw] OR thromboo* [tw] OR thrombop* [tw] OR thromboq* [tw] OR thrombor* [tw] OR thrombos* [tw] OR thrombot* [tw] OR thrombou* [tw] OR thrombov* [tw] OR thrombox* [tw] OR thromboy* [tw] OR thromboz* [tw] OR emboli* [tw] OR occlus* [tw]
5. #3 and #4
6. brain [tw] OR cerebro* [tw] OR cerebra* [tw] OR cerebell* [tw] OR intracerebral [tw] OR intracranial [tw] OR subarachnoid [tw]
7. haemorrhag* [tw] OR hemorrhag* [tw] OR haematoma* [tw] OR hematoma* [tw] OR bleed* [tw]
8. #6 and #7
9. brain injury [tw] OR brain injuries [tw] OR brain injured [tw]
10. hemipleg* [tw] OR hemipar* [tw] OR paresis [tw] OR paretic [tw] OR dystoni* [tw]
11. #1 OR #2 OR #5 OR #8 OR #9 OR #10
12. (“Self-assessed health” OR “Self-rated health” OR “Perceived health” OR “Self-evaluated health” OR “Self-reported health” OR “Self-ratings of health” OR “Self-assessments of health” OR “Self-perceptions of health” OR “Self-evaluated health” OR “Self-evaluations of health” OR “Self-evaluation of health”)
13. #11 AND #12

## Abbreviations

ADL: Activities of daily living
COOP/ WONCA: Primary Care Cooperative Information Project/ World Organization of National Colleges Academies
COPD: Chronic obstructive pulmonary disease
EQ5D: EuroQol 5D
EQtherm: EuroQol thermometer
EQVAS: EuroQol visual analog scale
LILACS: Latin American and Caribbean Health Sciences Literature
MEDLINE: Medical Literature Analysis and Retrieval System Online
PEDro: Physiotherapy Evidence Database
PRISMA: Preferred Reporting Items for Systematic Review and Meta-Analysis
PROSPERO: International Prospective Register of Systematic Reviews
QoL: Quality of life
SCIELO: Scientific Electronic Library Online
SF-12: Short Form Health Survey 12
SF-36: Short Form Health Survey 36
SRH: Self-rated health
TREND: Transparent Reporting of Evaluations with Nonrandomized Designs
WHO: World Health Organization
WHODAS 2.0: World Health Organization Disability Assessment Schedule
